# Prenatal and Early Life Exposure to Stressful Life Events and Risk of Autism Spectrum Disorders: Population-Based Studies in Sweden and England

**DOI:** 10.1371/journal.pone.0038893

**Published:** 2012-06-13

**Authors:** Dheeraj Rai, Jean Golding, Cecilia Magnusson, Colin Steer, Glyn Lewis, Christina Dalman

**Affiliations:** 1 Academic Unit of Psychiatry, School of Social and Community Medicine, University of Bristol, Bristol, United Kingdom; 2 Division of Public Health Epidemiology, Department of Public Health Sciences, Karolinska Institutet, Stockholm, Sweden; 3 Avon and Wiltshire Partnership Mental Health NHS Trust, Bristol, United Kingdom; 4 Centre for Child and Adolescent Health, School of Social and Community Medicine, University of Bristol, Bristol, United Kingdom; The University of Western Australia, Australia

## Abstract

**Background and Aim:**

Exposure to stressful life events during pregnancy has been suggested as a potential risk factor for offspring Autism Spectrum Disorders (ASD), but the literature is limited and inconsistent. We tested the hypothesis that maternal exposure to stressful life events would be associated with increased risks of offspring ASD, and that these risks would be highest for exposures during the prenatal period.

**Methods and Results:**

We used prospectively collected data from two large population based studies in Sweden and England. In the Swedish study of 4429 ASD cases and 43277 controls, our exposure comprised the occurrence of any severe life event before and during pregnancy and the child's early life. In the English study (maximum n = 11554, ASD n = 72), we studied the risk of offspring ASD in relation to a combined maternal exposure to multiple (up to 42) common and rare life events, as well as their perceived impact upon the mother during pregnancy and early life. In crude and adjusted regression analyses in both studies, we found no evidence of an association between prenatal life events, or their number and perceived impact and the risk of offspring ASD. Sub-group analysis of ASD with and without intellectual disability in the Swedish study yielded similar results.

**Conclusion:**

We found no evidence to support the hypotheses that exposure to stressful life events during the prenatal period is associated with an increased risk of offspring ASD.

## Introduction

Autism spectrum disorders (ASDs) are developmental disorders characterized by impairments in reciprocal social interaction, communication, restricted patterns of interests and stereotyped behaviours [1]. The reported prevalence of ASDs has risen steadily over the past few decades, and approximately 1% of the child population in the UK [1] and the USA [2] have recently been estimated to have ASDs [3]. ASDs are disabling conditions and associated with significant costs to individuals, their families and society [4] but their aetiology is not well understood. Although genetic factors are important, it is also acknowledged that environmental factors may play a role in the aetiology of ASD [5], [6].

Maternal exposure to stressful life events during pregnancy has been suggested as a potential risk factor for ASD [7], [8]. Animal experiments have found that exposure to stress during pregnancy may adversely affect the neurodevelopment of the offspring, including in domains relevant to autism (for review see Kinney et al, 2008) [7]. Mechanisms such as DNA methylation [9], or programming of the hypothalamic-pituitary-adrenal axis have been proposed as potential pathways by which psychological stress could affect neurodevelopment [7]. Although biologically plausible, the evidence supporting the relationship between psychological stress in pregnancy and ASD in human studies is limited and inconsistent.

Studies supporting the role of stressful events on the risk of ASD include an ecological investigation reporting that prenatal exposure to tropical storms was associated with a higher prevalence of ASD [10]; a small study (n = 56) finding a higher occurrence of ‘family discord’ reported by mothers of ASD children as compared to controls at antenatal interviews [11]; and a study based on maternal recall, showing that ASD mothers were more likely to report having stressful life events during pregnancy than controls [12]. Furthermore, a prospective Australian study, found a small but significant association between life-event exposure during pregnancy and autistic traits in 2 year old male, but not female children as measured by a subscale of the Child Behaviour Checklist [13]. In contrast, a large Danish study did not find evidence for an association between maternal exposure to bereavement during pregnancy and risk of offspring ASD [14]. Although based in the general population, this study had an unusually low cumulative incidence of ASD (0.16%), and may have been prone to outcome misclassification. The evidence on the role of stressful life events in the development of ASD is therefore inconclusive.

Methodologically strong studies on this issue are required, and may help provide a better understanding of the aetiology of ASD. We used two large population-based cohorts with prospectively collected data and complementary strengths to investigate related aspects of this research question. In the first study, based in Sweden, using record linkage data on almost 4500 individuals with ASD, we investigated the relationship between the occurrence of severe, but rare life events during pregnancy and offspring ASD. In the second study, based in England, we used data on the combined occurrence of over 40 common and rare life events along with their perceived impact, and studied these in relation to the risk of offspring ASD. Life events in both studies were measured at several time points, including pregnancy and early life. We examined the hypothesis that the exposures in both cohorts would be associated with an increased risk of offspring ASD, and that these risks would be highest when the exposures occurred during the prenatal period.

## Methods

### Study cohorts

#### The Stockholm Youth Cohort (SYC)

The SYC is a register based cohort of the total child population (aged 0–17 years) living in Stockholm County between 2001 and 2007 (n = 589,114) and comprises prospectively collected information on children and their first-degree relatives through record linkage with an extensive range of Swedish national and regional health and administrative registers [15]–[17]. The Research Ethics Committee at Karolinska Institutet, Stockholm, provided ethical approval for the analysis of anonymized record-linkage data in the cohort without individual consent.

#### The Avon Longitudinal Study of Parents and Children (ALSPAC)

ALSPAC is a longitudinal study established to explore the environmental, social, psychological and genetic factors associated with child health and development [18]. It recruited 14,541 pregnant women in the Bristol area of England who had an expected delivery date between April 1991 and December 1992, resulting in 14,062 live births. The study collected detailed information on mothers and children at repeated times during pregnancy and throughout childhood [18]. Ethical approval was obtained from the ALSPAC Law and Ethics Committee and the Southmead, Frenchay, UBHT and Weston Research Ethics Committees. Participating mothers consented to the use of anonymized linked data for research at the point of enrolment.

### Outcome ascertainment

#### ASDs in the Stockholm Youth Cohort

Children and young people with a diagnosis of an ASD (n = 5100, prevalence 0.9% in the full cohort) in the SYC have been identified using a multidisciplinary case ascertainment approach, via national and regional registers covering all pathways (including healthcare, social care and education) of ASD care in Stockholm County [15]–[17]. The Swedish system of child welfare clinics allows regular screening of children for developmental problems. Children suspected of autism undergo structured multidisciplinary assessments that include diagnostic evaluations covering the child's social, medical and developmental history by parental report, direct observation of the child, and a structured neuropsychiatric assessment including cognitive testing (using standardized tools appropriate for the child's age and developmental level) [19]. A case-note validation study on a randomly selected subset of 200 ASD cases in the SYC found 96% to be compatible with DSM-IV criteria of ASD [15], [17].

#### ASDs in the Avon Longitudinal Study of Parents and Children

Children diagnosed with ASD by age 11 (n = 86, prevalence 62/10,000) in ALSPAC have been identified through National Health Records and the Pupil Level Annual Schools Census (PLASC) [20]. A validation study conducted by a team of researchers, and led by an experienced consultant paediatrician confirmed these diagnoses in relation to ICD-10 criteria. The cases ascertained from health records were only considered as having an ASD if the diagnosis was made by a multidisciplinary team [20].

In line with the evidence supporting a dimensional view towards ASD [21], [22] and the DSM-V working group recommendations [21], we studied ASDs as a single group in both datasets [21]. In the SYC, we also dichotomized the ASD variable into two subgroups based on the presence or absence of recorded comorbid intellectual disabilities (ID) based on previous recommendations [22], since these groups may have partly different etiologies [15]. We did not apply this strategy to the ALSPAC ASD cases since these were relatively few.

### Exposures: Maternal life event measures

#### Life event measures in the Stockholm Youth Cohort

We used record linkage via national identity numbers to identify first-degree relatives of children in our study from the Swedish Multigenerational Register (http://www.scb.se/statistik/_publikationer/BE9999_2011A01_BR_BE96BR1102.pdf). We then linked their records with the Swedish National Cause of Death Register, The Swedish Cancer Registry (http://www.socialstyrelsen.se/register/halsodataregister/cancerregistret/inenglish), and The National Patient register (http://www.socialstyrelsen.se/register/halsodataregister/patientregistret/inenglish) in order to identify deaths, serious accidents (such as intracranial injuries) and serious illnesses (such as myocardial infarction, malignancies, full details below and in Table S1).

Using these sources, we identified the occurrence along with the date of:

- deaths (of any cause),
- serious accidents, injuries or events (ICD-10 codes S020-S021, S023-S024 S027-S029, S064-S069, S070-S079, T27-T28, T293, T297, T315-T319, T325-T329, T71, W75-W84, T780, T782, T802, T805, T809, T794, T811, T790, T791, T800, W65-W74, T751, X85-Y099, T74, Y35-Y36, T750, T754, X33, T36-T65, X40-49, S15, S25-S28, S35-S37, Y10-Y34, X60-X84, Y10-Y34, and equivalent ICD 8 and 9 codes), and
- diagnosis of life threatening or serious illnesses (ICD-10 codes, A39, B004, G00-G01, G04-G05, I21-I22, I46, I260, I269, O88, I60-I64, O873, O225, C00-C75, C81-C97 and equivalent codes for ICD 8 and 9) in these relatives (Table S1).

We extracted and coded these events in relation to the time of pregnancy. For our main analysis, we defined dichotomous exposure variables representing the presence or absence of any of the above life events in the year before birth, during pregnancy and during 1, 2 and 3 completed years after birth.

#### Life event measures in the Avon Longitudinal Study of Parents and Children

In ALSPAC, we used data collected from study mothers on over 40 different common and rare life events based on items suggested by Brown and Harris [23], Barnett et al [24] and Honnor et al [25] at 6 time points covering the prenatal period until 3 years after the child's birth. Details of the life event questions are available in Table S2. The questionnaires were administered at approximately 18 weeks gestation (covering events from beginning of pregnancy), 8 weeks postnatal (covering events from mid-pregnancy), 8 months postnatal (covering events since birth), 21-months postnatal (covering events since child was 8 months old), 33-months postnatal (covering events since child was one and a half years old), and at 3 yr 11 months postnatal (covering events since child was two and a half years old).

Each life event had 5 response categories indicating whether or not the event occurred and how greatly the respondent was affected by it. Each life event, at each time point was thus coded 0 if the event did not occur, 1 if event occurred but mother reported not being affected by event, 2 if event occurred and mother reported being minimally affected by it, 3 if event occurred and mother reporting being moderately affected by it, and 4 if event occurred and mother reported being severely affected by it. For our main analysis, we derived a weighted life events score for each of the 6 time points by adding the above values for each of the life events studied. We used principal factor analysis of the individual life event questions to investigate the latent weighted life-event exposure trait. The factor scores were highly correlated (r>0.85 at each time point) with the corresponding weighted life event scores. We also categorised the weighted life event score into a binary variable using a 75^th^ percentile cut-off (i.e. top quartiles of score vs. lower three quartiles). We also made a separate variable only including the total number of life events, regardless of their perceived impact.

### Statistical Methods

Analyses were conducted using Stata Version 10.1 for Windows.

#### The Stockholm Youth Cohort

For each individual case of ASD we chose 10 living controls matched by date of birth and gender. Controls were from within the SYC without the diagnosis of ASD at the time of case ascertainment. We excluded adopted children, those not living in Stockholm County for at least 4 years and those with missing data.

After descriptive analyses, we used conditional logistic regression models to study the relationship between the occurrence of any life event (in the year before pregnancy, during pregnancy and within 1, 2 and 3 completed years after birth), and an ASD diagnosis in the child. We repeated these analyses after dichotomizing the outcome into ASD with and without intellectual disability. In adjusted models we first (Model 1) controlled for age of mother and father (continuous variables) at birth of child, parity (0,1,2,3 or more), quintiles of family disposable income adjusted for family size, highest educational qualification of mother or father (primary school or less, secondary school, university or higher education), occupational class (highest of mother or father), migration status of parents (mother born in Sweden, Europe, North America, Africa or Asia and Oceania); these variables were chosen based upon their association with ASD in previous studies [15], [17], [26], [27]. In an additional model (Model 2) we further adjusted for life events at other time points to identify sensitive or critical periods in any observed relationships.

#### ALSPAC

In ALSPAC, we first investigated whether non-participation in subsequent waves of questionnaires was associated with exposure to life events at baseline, or predicted the outcome of an ASD diagnosis in the child.

After descriptive analyses, we used logistic regression models to investigate the associations between the weighted life events variables at each of the 6 time points under study, and a diagnosis of ASD. We adjusted for (Model 1) maternal and paternal age (continuous variables), highest occupational class of either parent (UK Registrar General's classification I/II, III manual/non-manual, IV/V), highest educational qualification of either parent (UK Degree education, A levels, O levels, or vocational or no educational qualifications), tenure of accommodation (own/mortgaged property, rented privately, social housing), parity (0, 1, 2 or more) and sex of child. We then further adjusted the above model (Model 2) for the weighted life event scores at all other time points under study. We repeated the above analyses i) using a binary variable representing the top quartile vs. the lower 3 quartiles of weighted life events score as the independent variable, ii) using the total number of life events, regardless of their effect on the mother as the independent variable, and iii) on a complete case cohort with complete data at all time points.

## Results

### The Stockholm Youth Cohort

We included 4429 children with ASD (1828 had a co-morbid intellectual disability, 2601 did not) and 43277 controls with complete data in our analysis. Table 1 shows the proportion of ASD cases and controls whose mothers experienced a life event at each time point studied in relation to birth. The exposure to the severe stressful life events studied was rare, less than 1% at most time points, and largely similar in cases and controls at the time points studied except the 3^rd^ year of the child's life, where mothers of ASD children were more likely to experience significant life events (Table 1).

**Table 1:** Descriptive statistics for cases and controls experiencing a serious life event in the Stockholm Youth Cohort.

	All Autism Spectrum Disorders (ASD)			ASD without intellectual disability			ASD with intellectual disability		
Timing of Stressful life event	Cases n = 4429 N(%)	Controls n = 43277 N (%)	P*	Cases n = 2601 N(%)	Controls n = 24986 N(%)	P*	Cases n = 1828 N(%)	Controls n = 18291 N(%)	P*
**Year before** **pregnancy**	41 (0.93)	317 (0.73)	0.135	23 (0.88)	191 (0.76)	0.455	18 (0.98)	126 (0.69)	0.147
**During** **pregnancy**	17 (0.38)	164 (0.38)	0.992	13 (0.50)	95 (0.38)	0.403	4 (0.22)	69 (0.38)	0.290
**1st year after** **birth**	29 (0.65)	228 (0.53)	0.242	18 (0.69)	139 (0.56)	0.350	11 (0.60)	89 (0.49)	0.481
**2nd year after** **birth**	30 (0.68)	252 (0.58)	0.391	22 (0.85)	144 (0.58)	0.103	8 (0.44)	108 (0.59)	0.497
**3rd year after** **birth**	38 (0.86)	247 (0.57)	0.012	19 (0.73)	133 (0.53)	0.138	19 (1.04)	114 (0.62)	0.038

Footnote:

* p values derived from conditional logistic regression (cases and controls matched by age and sex).

Individuals can fall in more than one row if they experience life events at more than one time point.

In logistic regression analysis conditioned on age and sex, we found no evidence of any relationships between prenatal life events and offspring autism spectrum disorders as a group in either crude or adjusted models (Table 2). In contrast, there appeared to be an association between life events occurring between 2–3 years of the child's life and a diagnosis of ASD (p = 0.012). We found similar results when ASDs were grouped by intellectual disability, although point estimates of risk when life events occurred during pregnancy were higher for ASD without intellectual disability [OR 1.21 95% CI (0.67–2.20)] than those for ASD with intellectual disability [OR 0.47 95% CI (0.17–1.31), Table 3]. Sensitivity analysis using only bereavement as the exposure also yielded results similar to our main analysis (data not shown).

**Table 2:** Relationship between maternal exposure to life events and risk of offspring ASD in the Stockholm Youth Cohort.

	All Autism Spectrum Disorders		
Timing of Stressful life event	Crude OR (95% CI)	Adjusted OR Model 1* (95% CI)	Adjusted OR Model 2# (95% CI)
Year before pregnancy	1.28 (0.92–1.79)	1.28 (0.91–1.78)	1.28 (0.91–1.78)
During pregnancy	1.00 (0.60–1.65)	0.89 (0.54–1.48)	0.88 (0.53–1.46)
1st year after birth	1.26 (0.85–1.87)	1.23 (0.83–1.82)	1.22 (0.82–1.82)
2nd year after birth	1.18 (0.81–1.73)	1.09 (0.74–1.61)	1.06 (0.72–1.56)
3rd year after birth	1.56 (1.10–2.20)	1.52 (1.08–2.16)	1.51 (1.07–2.14)

Legend: Conditional logistic regression models estimating association of the mother's experience of a serious life event and risk of offspring ASD in the Stockholm Youth Cohort.

Footnote:

OR = Odds Ratio, 95% CI = 95% Confidence Interval.

* Model 1: adjusted for age of mother and father at birth of child, parity, quintiles of family disposable income adjusted for family size, highest educational qualification of mother or father, occupational class (highest of mother or father), migration status of parents.

# Model 2: Model 1 further adjusted for occurrence of life events at other time points under study.

**Table 3:** Relationship between maternal exposure to life events and risk of offspring ASD with or without intellectual disability in the Stockholm Youth Cohort.

	ASD without intellectual disability			ASD with intellectual disability		
Timing of Stressful life event	Crude OR (95% CI)	Adjusted OR Model 1* (95% CI)	Adjusted OR Model 2# (95% CI)	Crude OR (95% CI)	Adjusted OR Model 1* (95% CI)	Adjusted OR Model 2# (95% CI)
**Year before pregnancy**	1.18 (0.76–1.83)	1.23 (0.79–1.93)	1.21 (0.78–1.89)	1.45 (0.88–2.41)	1.39 (0.83–2.32)	1.39 (0.83–2.33)
**During pregnancy**	1.28 (0.72–2.30)	1.26 (0.69–2.27)	1.21 (0.67–2.20)	0.58 (0.21–1.59)	0.47 (0.17–1.31)	0.47 (0.17–1.31)
**1st year after birth**	1.27 (0.77–2.08)	1.25 (0.75–2.06)	1.21 (0.73–2.00)	1.26 (0.67–2.37)	1.16 (0.61–2.23)	1.19 (0.62–2.27)
**2nd year after birth**	1.46 (0.93–2.30)	1.40 (0.88–2.21)	1.34 (0.84–2.13)	0.78 (0.38–1.61)	0.70 (0.33–1.45)	0.68 (0.32–1.42)
**3rd year after birth**	1.44 (0.89–2.35)	1.46 (0.89–2.39)	1.41 (0.86–2.32)	1.69 (1.03–2.76)	1.62 (0.99–2.68)	1.63 (0.99–2.69)

Legend: Conditional logistic regression models estimating association of mother's experience of a serious life event and risk of ASD with and without intellectual disability in the Stockholm Youth Cohort.

Footnote:

OR = Odds Ratio, 95% CI = 95% Confidence Interval.

* Model 1: adjusted for age of mother and father at birth of child, parity, quintiles of family disposable income adjusted for family size, highest educational qualification of mother or father, occupational class (highest of mother or father), migration status of parents.

# Model 2: Model 1 further adjusted for occurrence of life events at other time points under study.

### ALSPAC

The highest response rate to the life events questionnaire was in the 8 week postnatal questionnaire (11554 complete responses) covering the life events from mid-pregnancy to two-months postnatally, and the lowest in the 3 year 11 months questionnaire (n = 9616) (Table 4). Non-responders in all subsequent time points studied after the 8 week postnatal questionnaire were more likely to have been in the top quartile of weighted life event scores at baseline (*X^2^* 20.270, d.f = 1, p<0.001) and 8 week postnatal questionnaire (*X^2^* 8.948, d.f = 1, p = 0.003). The non-respondents were no more likely to have a child subsequently diagnosed with ASD (*X^2^* 0.650, d.f = 1, p = 0.420).

**Table 4:** Descriptive statistics of life event exposures in mothers of children with and without ASD in ALSPAC.

		Median (Inter-quartile range) of weighted life events score			Percentage in top quartile of weighted life event score (%)		
Timing of stressor	N (ASD/no ASD)	Mothers of children with ASD	Mothers of children without ASD	P*	Mothers of children with ASD	Mothers of children without ASD	P# (×^2^)
**Early Pregnancy-18 weeks**	73/11153	6 (3,10)	6 (3,12)	0.176	19.2	22.6	0.489
**Mid Pregnancy-2 month post delivery**	70/11484	6 (3,12)	6.5 (3,11)	0.752	20.0	22.4	0.628
**Birth- 8 months**	72/11177	8 (4.5,13)	8 (4,14)	0.994	13.9	22.5	0.081
**8–21 months**	69/10311	7 (3,14)	9 (4,15)	0.035	15.9	25.0	0.084
**18 m–33 months**	65/9655	9 (4,15)	11 (5,18)	0.082	13.9	22.9	0.082
**2.5 yr to 3 yr 11 months**	64/9552	8.5 (4,14)	9 (4,17)	0.152	14.1	23.1	0.088

Legend: Descriptive statistics of weighted life event scores (incorporating the number and perceived impact of life events) in mothers of children with and without ASD at the time points studied in ALSPAC.

Footnote:

* P value obtained after square root transformation of weighted life event score to approximate normal distribution.

# Chi square p-value (d.f. = 1).

The exposure variable studied in ALSPAC included both rare and common life events (Table S2), and the majority of the mothers at any time point studied reported having experienced one or more of the life events in the questionnaires. Descriptive statistics for the prevalence of individual life events in the sample and their perceived impact are presented in Table S3. Table 4 contains descriptive statistics for the weighted life events score at each time point studied.

There was no evidence for an association between prenatal weighted life events score (incorporating the number and severity of life events) and a diagnosis of ASD in the ALSPAC data in either crude or adjusted logistic regression models (Table 5). Analyses after dichotomizing the exposure variable into the top quartile of the weighted life events score vs. bottom 3 quartiles also yielded similar results (Table 6), although the associations between life events during the prenatal period and ASD appeared to increase when life events at other time points were accounted for in the regression model [for exposure in early pregnancy OR 1.25 95% CI (0.54–2.89), and late pregnancy OR 1.45 95% (0.61–3.44)].

**Table 5:** Relationship between maternal life event scores (continuous variable) and offspring ASD in ALSPAC.

	Autism Spectrum Disorder		
Timing of stressor	Crude OR (95% CI)	Adjusted OR Model 1* (95% CI)	Adjusted OR Model 2# (95% CI)
**Early Pregnancy-18w**	0.98 (0.95–1.01)	0.99 (0.95–1.03)	1.00 (0.95–1.06)
**Mid Pregnancy-2 months post delivery**	0.99 (0.95–1.02)	0.98 (0.94–1.02)	1.01 (0.95–1.07)
**Birth- 8 months**	0.99 (0.96–1.02)	0.98 (0.95–1.02)	1.02 (0.96–1.07)
**8–21 months**	0.97 (0.93–1.00)	0.96 (0.93–1.00)	0.97 (0.92–1.03)
**18 m–33 months**	0.97 (0.94–1.00)	0.97 (0.94–1.00)	0.99 (0.94–1.03)
**2.5 yr to 3 yr 11 months**	0.98 (0.95–1.01)	0.97 (0.94–1.01)	0.96 (0.91–1.01)

Legend: Logistic regression models to study the association of maternal weighted life event scores (as a continuous variable) and offspring ASD at the time points studied in ALSPAC.

Footnote:

OR = Odds Ratio, 95% CI = 95% Confidence Interval.

* Model 1: adjusted for maternal and paternal age, occupational class (highest or either parent), educational qualification (highest of either parent), tenure of accommodation, parity and sex of child.

# Model 2: Model 1 further adjusted for the weighted life event scores at all other time points under study.

**Table 6:** Relationship between the maternal life event scores (top quartile) and offspring ASD in ALSPAC.

	Autism Spectrum Disorder		
Timing of stressor	Crude OR (95% CI)	Adjusted OR Model 1* (95% CI)	Adjusted OR Model 2# (95% CI)
**Early Pregnancy-18w**	0.81 (0.45–1.46)	0.97 (0.51–1.85)	1.25 (0.54–2.89)
**Mid Pregnancy-2 months post delivery**	0.86 (0.48–1.56)	0.89 (0.46–1.73)	1.45 (0.61–3.44)
**Birth- 8 m**	0.56 (0.28–1.08)	0.41 (0.18–0.98)	0.45 (0.14–1.38)
**8–21 m**	0.57 (0.30–1.09)	0.58 (0.28–1.18)	1.03 (0.43–2.46)
**18 m–33 m**	0.54 (0.27–1.09)	0.59 (0.28–1.27)	0.89 (0.33–2.31)
**2.5 yr to 3 yr 11 m**	0.54 (0.27–1.01)	0.50 (0.22–1.01)	0.29 (0.08–1.00)

Legend: Logistic regression models to study associations of mothers in the top quartile of weighted life event scores and offspring ASD at the time points studied in ALSPAC.

Footnote:

OR = Odds Ratio, 95% CI = 95% Confidence Interval.

* Model 1: adjusted for maternal and paternal age, occupational class (highest or either parent), educational qualification (highest of either parent), tenure of accommodation, parity and sex of child.

# Model 2: Model 1 further adjusted for the weighted life event scores at all other time points under study.

We repeated the above analyses using the total number of life events as the independent variable, irrespective of their perceived effect on the mother and found results similar to those reported above (data not shown). Analyses conducted on a dataset with complete information on life events and covariates at all time points also led to similar results (data not shown).

## Discussion

We used prospectively collected data from two large population-based studies in Sweden and England to study related aspects of the relationship between prenatal stressful life events and risk of offspring autism spectrum disorders. In the Swedish study, our exposure comprised the occurrence of any severe, but relatively rare life event before and during pregnancy and early life. In the English study, we studied the combined exposure to multiple life events, both common and rare, along with their perceived impact upon the mother during pregnancy and the child's early life. We tested the hypothesis that exposure to stressful life events, as well as their combined occurrence and perceived impact would be associated with increased risk of offspring ASD, and that these risks would be highest for exposures during the prenatal period. We found no evidence to support the hypotheses in either cohort. These findings were consistent in all analyses and for ASD children with or without comorbid intellectual disability.

### Strengths and Limitations

Both sources of data we used are large population-based intergenerational cohorts with prospectively recorded data on exposures and a range of potential confounders, minimising the possibility of selection and recall bias and confounding. The SYC analysis included the largest number of ASD cases studied in this context (n = 4429), with almost complete follow up. Life events studied in the SYC were typically severe and rare. This was complemented by information on a wide range of potentially stressful life events (Table S2 and S3), along with their perceived impact on the mother in ALSPAC, a potentially overlooked dimension of this topic. Several common life events were perceived to be as stressful as rare events such as bereavement (Table S3). We weighted the life events according to maternal responses which have good face validity, but which we are unable to compare with any measures of biological stress response; however, we are not aware of any population-based studies with such data. The results were similar when we disregarded such weighting of perceived stress, using the more widely used approach of summing the life events [13].

Although both our cohorts were large; the stressful life events studied in the Stockholm Youth Cohort were experienced by less than 1% of the population. And while we had data on the presence of more common life events, their number and perceived severity in ALSPAC, the ASD cases were relatively few. In a retrospective calculation, we estimated that in the SYC, with 4429 cases and 47706 controls, we had 80% power at the 5% significance level to detect OR's of 1.54 for an exposure of 0.75% and 1.79 for exposure 0.38%. In ALSPAC, N = 11153 (73 ASD cases) we had 80% power at the 5% significance level to detect an OR of 1.96 for the top quartile life-event score exposure, and OR of 2.05 in N = 9552 (64 ASD cases). Therefore, the possibility of null findings due to a lack of statistical power cannot be excluded. Further, the upper limits of the confidence intervals of our associations cannot exclude the possibility of a marginally increased risk of ASD related to exposure to the life events, such as those observed in a recent study of autistic traits [13].

A selective attrition in ALSPAC has previously been established, and mothers with socioeconomic disadvantages were more likely to drop out from the study [28]. There was evidence that mothers with higher weighted life-events were less likely to respond to further questionnaires after the initial two time points under study. However, non-response was not associated with the future diagnosis of ASD in the child. Attrition based upon exposure characteristics is unlikely to have any significant impact upon the results of our regression analysis, and it was reassuring that there was no evidence of differential attrition based upon our outcome (eventual offspring ASD case status) which would be more likely to introduce biased estimates [28]. Furthermore, we controlled for several socioeconomic characteristics that are known to be associated with attrition in ALSPAC, which would have further minimized the possibility of such bias [28].

### Comparison with previous studies

There are two population-based studies with which to compare our findings [13], [14]. Li et al [14], used data from Danish Registries and found no significant relationships between the experience of bereavement by mothers during pregnancy and risk of offspring ASDs. Although the findings of our Swedish study were identical to this study, some methodological differences are notable. A majority of the ASD cases in the Danish study were ascertained using inpatient registries with a 0.16% cumulative incidence of ASD (end of follow up 2006). This is much lower than contemporary reports [1], [2], and probably explained by the fact that the majority of children with ASD would not require inpatient care (the main source for case ascertainment), making outcome misclassification possible. The SYC, by using a multisource method of case ascertainment is likely to have captured the majority of the ASD cases diagnosed in Stockholm County. Furthermore, Li et al studied bereavement as the marker of prenatal stress; and we studied, apart from bereavement, a larger range of significant and serious events that may be equally stressful. Finally, in contrast with the Danish study, the SYC only had record linkages with details of the offspring's first degree relatives and therefore, we were unable to study the exposures in relation to events occurring in other relatives of the mother. The second study used prospective data from the Western Australia Pregnancy Cohort (Raine) with a design similar to ALSPAC. This study investigated the association of the sum of 10 life events collected from pregnant women at 18 and 34 weeks of gestation, and offspring autistic traits measured by the Pervasive Developmental Problems (PDP) subscale of the Child Behaviour Checklist (n = 1644) [13]. The number of maternal life events during pregnancy were associated with autistic traits in the offspring in male but not female children after controlling for a number of covariates, explaining 1% of the variance in autistic trait scores. We did not find such an effect for diagnosed ASD in ALSPAC, which may be explained by either the modest specificity (42%) of the PDP subscale in detecting autism as compared to the gold-standard ADOS-G [13], or due to the small number of diagnosed ASD cases in ALSPAC resulting in insufficient power to detect such a marginally increased risk.

Our findings are also not consistent with the three other studies on this topic that reported a positive association between prenatal stressful events and ASD, but these had significant methodological limitations. Kinney et al, using weather services data in the USA, found a greater prevalence of ASD in cohorts that experienced tropical storms particularly during mid-late pregnancy [10]. Although a thoughtfully designed study, using exposure to storms as a natural experiment mimicking a randomized trial, the ecological design, due to absence of individual-level data could not allow for a robust conclusion regarding the role of prenatal stress in the aetiology of ASDs [29]. Ward retrospectively reviewed antenatal interview records of mothers of 56 autism cases and healthy controls (studied 13 years after the original study of cases) and reported a significantly greater prevalence of ‘family discord’ in the autism mothers as compared to controls [11]. This study was prone to measurement bias since the author had to make an unblinded judgement on the presence of ‘family discord’, and conducted the review of control subjects several years after the original study. Since the study recruited a small number of participants from a single clinic, this study was also prone to selection bias. Furthermore, no adjustment was made for potential confounding. Finally, a study that sent out questionnaires to parents of children with autism (mean age 7.9 years), Down syndrome (mean age 9 years) and healthy controls (mean age 10.2 years) reported that the mothers of children with autism recalled experiencing a significantly higher number of life events particularly during 21–32 weeks of pregnancy. This study was unable to adjust for confounding, and was prone to recall and attribution bias The authors concluded that their data were ‘far too preliminary to suggest causative relationships’ and called for prospective and more comprehensive studies on this topic [12].

The associations of life events occurring at different time points in relation to pregnancy and the child's life were similar at all time points under study bar some exceptions. In the SYC, there appeared an almost 50% increase in risk with the occurrence of a severe life event between two and three years of the child's life. The meaning of this finding is unclear, especially since it was not corroborated in the ALSPAC analysis. If not a chance association, reverse causality may be an explanation, since the first signs of ASD are apparent at this age and may add an extra burden of stress, making the family prone to life events. Another observation worthy of cautious discussion was the finding of an inflated association between mothers with the highest weighted life events during last half of pregnancy and first two months after birth and offspring ASD in ALSPAC after adjusting for life event scores at other time points (Table 6, Model 2 compared with Model 1). Stress during middle-late pregnancy, when synaptogenesis in the human brain begins has been hypothesised to be a sensitive period when neurodevelopmental disruption may lead to the development of ASD [7]. However, it is impossible to make any robust conclusions about the significance of this finding due to the wide confidence intervals, and since this finding was not observed in the SYC data.

We have previously posited the likelihood of prenatal stress in consequence of studies conducted within the Stockholm Youth Cohort, investigating the relationship between maternal migration [15] and socioeconomic status respectively [17], and the risk of ASD. In the first study, we found maternal migration, particularly during pregnancy, and from areas of low human development (as a possible indicator of stressful migration of asylum seeking population in Sweden) to be associated with a higher risk of ASD with comorbid intellectual disabilities [15]. In the second study, we found measures of a lower prenatal socioeconomic status of parents to be associated with a higher risk of ASD [17]. The results of both these studies may be consistent with the stress hypothesis, but alternative explanatory mechanisms could also be at play [15], [17]. If stress was the explanation, it should be noted that both stressful migration (and subsequent period of settlement) and socioeconomic stressors are likely to be relatively chronic stressors, it is therefore possible that the acute life events included in the present study do not have sufficient or sustained enough effects to impair foetal neurodevelopment.

### Conclusions and Future Directions

We did not find evidence of a significant relationship between stressful life events during pregnancy and offspring autism spectrum disorders in these two large population based cohorts. There are four alternative explanations for these findings that future studies may help clarify. First, despite being large population-based studies, it is possible that exposure to life events may confer a low increased risk of ASD that could not be detected in either of our samples, or in a previous large Danish study [14] due to a lack of statistical power. Clarification of this issue would require replication of our studies as larger ASD cohorts with relevant data become available. The second possibility is that chronic and sustained, but not acute stressors influence neurodevelopment and increase the risk of ASD, and future studies should explore other conceptual models of stress using a variety of exposures. Third, it is possible that acute or chronic stressors have differential relationships with the various dimensional traits that make up the concept of autism spectrum disorders, as has been highlighted by a recent study [13], and these relationships could be tested in cohorts with relevant data. Finally, there may not be a link between maternal exposure to stress and risk of ASD and until robust evidence is available, caution should be exercised before concluding that stressful life events experienced during pregnancy confer an increased risk of offspring ASD.

## Supporting Information

Table S1 **Diagnostic categories for exposures according to main diagnoses in the Swedish National Patient Register, the Swedish Cancer Register and the Swedish Cause of Death Register.**
(DOC)

Table S2 **This is an example of questions asked in the 18 week life event questionnaire in ALSPAC.** The questionnaires were similar across other the time points studied but had relevant minor changes in time points after the birth of the index child (for example, you were bleeding and thought you might miscarry was later changed to ‘you had a miscarriage’ for future pregnancies). The question was worded as *“Listed below are a number of events which may have brought changes in your life. Have any of these occurred since (time period covered, in the 18 week questionnaire this was: since you became pregnant)? If so, please assess how much effect it had on you.”*
(DOC)

Table S3 **Prevalence of individual life events reported by ALSPAC mothers at 18 weeks gestation, and their mean perceived severity.**
(DOC)
