# Supplementary material for: Prenatal and Early Life Exposure to Stressful Life Events and Risk of Autism Spectrum Disorders: Population-Based Studies in Sweden and England
Source: PLoS One. 2012 Jun 13;7(6):e38893. doi: 10.1371/journal.pone.0038893 (PMC3374800; doi:10.1371/journal.pone.0038893)
Supplement: Table S1 — Diagnostic categories for exposures according to main diagnoses in the Swedish National Patient Register, the Swedish Cancer Register and the Swedish Cause of Death Register. (DOC) [file pone.0038893.s001.doc]

Table S1. Diagnostic categories for exposures in the Stockholm Youth Cohort

|  | ICD8 in 1973-1986 | ICD9 in 1987-1996 | ICD10 in 1997-2006 |
| --- | --- | --- | --- |
| **Death of a parent and sibling** |  |  |  |
| Death caused by suicide (including undetermined intent)  Death caused by accident | E950-E959, E980-E989 | E950-E959, E980-E989 | X60-X84, Y10-Y34 |
| Death by all other causes |  |  |  |
| **Serious illness of a parent and sibling** |  |  |  |
| Meningitis | 320-323, 036, 054,04 | 320-323, 036, 054D | A39, B004, G00-G01, G04-G05 |
| Myocardial infarction, cardiac arrest | 410 | 410, 427F | I21-I22, I46 |
| Pulmonary embolism | 450, 673 | 415B, 673 | I260, I269, O88 |
| Cerebral infarction, cerebral haemorrhage, stroke (NOS) | 430-34,36,37, 674,99 | 430-34,36,37, 671F, 674A | I60-I64, O873, O225 |
| Malignant neoplasms | 140-165, 170-175, 180-194, 200-209 | 140-175, 179-194, 200-209 | C00-C75, C81-C97 |
| **Serious injury/accident of a parent and sibling** |  |  |  |
| Intracranial injury, fracture of skull and facial bones , Crushing injuries to head and face | 800-801, 803-804, 852-854 | 800-801, 803-804, 852-854 | S020-S021, S023-S024 S027-S029, S064-S069, S070-S079 |
| Burns and corrosions of respiratory tract, other internal organs, and of >49% of body surface | 946-948 | 946-947, 948FGHWX | T27-T28, T293, T297, T315-T319, T325-T329 |
| Asphyxiation and other accidental threats to breathing | 994,7, E913, E911, E912 | 994H, E913, E911, E912 | T71, W75-W84 |
| Anaphylactic shock, certain reactions to transfusion, infusion, therapeutic injection | 782,9, 999,4, | 995A, 999E, 999W | T780, T782, T802, T805, T809 |
| Traumatic chock | 995,5, 999,8 | 958E, 998A | T794, T811 |
| Complications of trauma or transfusion, infusion, therapeutic injection; air embolism, fat embolism | 995,00, 995,10, 998,91 | 958A, 958B, 999B | T790, T791, T800 |
| Accidental drowning and submersion | 994,1; E910 | 994B, E910 | W65-W74, T751 |
| Assault, maltreatment syndromes | E960-E969 | E960-E969, 995F | X85-Y099, T74 |
| Legal intervention and operations of war | E970-E979, E990-E999 | E976-E977, E997-E999 | Y35-Y36 |
| Shock from lightning or electric current | 994,00, 994,80, E907 | 994A, 994W, E907 | T750, T754, X33 |
| Poisoning and toxic effects | 960-989, E859, E869, E877 | 960-989, E859, E866 | T36-T65, X40-49 |
| Injury of internal organs and blood vessels | 860-869 | 860-869, 900-902 | S15, S25-S28, S35-S37 |
| Event of undetermined intent* | E980-E989 | E980-E989 | Y10-Y34 |
| **Suicide attempt** |  |  |  |
| Suicide attempts**(intentional self-harm and event of undetermined intent) | E950-E959, E980-E989 | E950-E959, E980-E989 | X60-X84, Y10-Y34 |

* Only considered if the individual with this diagnosis is <7 years old.

** Only considered as suicide attempt if the individual with this diagnosis is >7 years old.
