# Supplementary material for: Prenatal and Early Life Exposure to Stressful Life Events and Risk of Autism Spectrum Disorders: Population-Based Studies in Sweden and England
Source: PLoS One. 2012 Jun 13;7(6):e38893. doi: 10.1371/journal.pone.0038893 (PMC3374800; doi:10.1371/journal.pone.0038893)
Supplement: Table S2 — This is an example of questions asked in the 18 week life event questionnaire in ALSPAC. The questionnaires were similar across other the time points studied but had relevant minor changes in time points after the birth of the index child (for example, you were bleeding and thought you might miscarry was later changed to ‘you had a miscarriage’ for future pregnancies). The question was worded as “Listed below are a number of events which may have brought changes in your life. Have any of these occurred since (time period covered, in the 18 week questionnaire this was: since you became pregnant)? If so, please assess how much effect it had on you.” (DOC) [file pone.0038893.s002.doc]

**Table S2: Life events ascertainment in ALSPAC**

|  | **Yes and affected me a lot** | **Yes, moderately affected** | **Yes, mildly affected** | **Yes, but did not affect me at all** | **No, did not happen** |
| --- | --- | --- | --- | --- | --- |
| 1. Your partner died | 4 | 3 | 2 | 1 | 0 |
| 2. One of your children died | 4 | 3 | 2 | 1 | 0 |
| 3. A friend or relative died | 4 | 3 | 2 | 1 | 0 |
| 4. One of your children was ill | 4 | 3 | 2 | 1 | 0 |
| 5. Your partner was ill | 4 | 3 | 2 | 1 | 0 |
| 6. A friend or relative was ill | 4 | 3 | 2 | 1 | 0 |
| 7. You were admitted to hospital | 4 | 3 | 2 | 1 | 0 |
| 8. You were in trouble with the law | 4 | 3 | 2 | 1 | 0 |
| 9. You were divorced | 4 | 3 | 2 | 1 | 0 |
| 10. You found that your partner didn't want your child | 4 | 3 | 2 | 1 | 0 |
| 11. You were very ill | 4 | 3 | 2 | 1 | 0 |
| 12. Your partner lost his job | 4 | 3 | 2 | 1 | 0 |
| 13. Your partner had problems at work | 4 | 3 | 2 | 1 | 0 |
| 14. You had problems at work | 4 | 3 | 2 | 1 | 0 |
| 15. You lost your job | 4 | 3 | 2 | 1 | 0 |
| 16. Your partner went away | 4 | 3 | 2 | 1 | 0 |
| 17. Your partner was in trouble with the law | 4 | 3 | 2 | 1 | 0 |
| 18. You and your partner separated | 4 | 3 | 2 | 1 | 0 |
| 19. Your income was reduced | 4 | 3 | 2 | 1 | 0 |
| 20. You argued with your partner | 4 | 3 | 2 | 1 | 0 |
| 21. You had arguments with your family or friends | 4 | 3 | 2 | 1 | 0 |
| 22. You moved house | 4 | 3 | 2 | 1 | 0 |
| 23. Your partner hurt you physically | 4 | 3 | 2 | 1 | 0 |
| 24. You became homeless | 4 | 3 | 2 | 1 | 0 |
| 25. You had a major financial problem | 4 | 3 | 2 | 1 | 0 |
| 26. You got married | 4 | 3 | 2 | 1 | 0 |
| 27. Your partner hurt your children physically | 4 | 3 | 2 | 1 | 0 |
| 28. You attempted suicide | 4 | 3 | 2 | 1 | 0 |
| 29. You were convicted of an offence | 4 | 3 | 2 | 1 | 0 |
| 30. You were bleeding and thought you might miscarry | 4 | 3 | 2 | 1 | 0 |
| 31. You started a new job | 4 | 3 | 2 | 1 | 0 |
| 32. You had a test to see if your baby was abnormal | 4 | 3 | 2 | 1 | 0 |
| 33. You had a result on a test that suggested your baby might not be normal | 4 | 3 | 2 | 1 | 0 |
| 34. You were told that you were going to have twins | 4 | 3 | 2 | 1 | 0 |
| 35. You heard that something that had happened might be harmful to the baby | 4 | 3 | 2 | 1 | 0 |
| 36. You tried to have an abortion | 4 | 3 | 2 | 1 | 0 |
| 37. You took an examination | 4 | 3 | 2 | 1 | 0 |
| 38. Your partner was emotionally cruel to you | 4 | 3 | 2 | 1 | 0 |
| 39. Your partner was emotionally cruel to your children | 4 | 3 | 2 | 1 | 0 |
| 40. Your house or car was burgled | 4 | 3 | 2 | 1 | 0 |
| 41. You had an accident | 4 | 3 | 2 | 1 | 0 |
| 42. a) Is there anything else which is not on the list which has concerned you or required additional effort from you to cope since becoming pregnant? If yes b) please describe........................  c) How did this affect you? (5 choices as for above questions) | 4 | 3 | 2 | 1 | 0 |
